# Rapid progression and mortality of lysosomal acid lipase deficiency presenting in infants

**DOI:** 10.1038/gim.2015.108

**Published:** 2015-08-27

**Authors:** Simon A. Jones, Vassili Valayannopoulos, Eugene Schneider, Stephen Eckert, Maryam Banikazemi, Martin Bialer, Stephen Cederbaum, Alicia Chan, Anil Dhawan, Maja Di Rocco, Jennifer Domm, Gregory M. Enns, David Finegold, J. Jay Gargus, Ornella Guardamagna, Christian Hendriksz, Iman G. Mahmoud, Julian Raiman, Laila A. Selim, Chester B. Whitley, Osama Zaki, Anthony G. Quinn

**Affiliations:** 1 Manchester Centre for Genomic Medicine, St. Mary's Hospital, CMFT, University of Manchester, Manchester, UK; 2 Hôpital Necker—Enfants Malades, Paris, France; 3 Synageva BioPharma Corp., Lexington, Massachusetts, USA; 4 New York-Presbyterian Hospital, New York, New York, USA; 5 North Shore LIG Health System, Manhasset, New York, USA; 6 University of California–Los Angeles, Los Angeles, California, USA; 7 University of Alberta, Edmonton, Alberta, Canada; 8 King's College Hospital NHS Foundation Trust, London, UK; 9 Istituto Giannina Gaslini-Ospedale Pediatrico, Genoa, Italy; 10 Monroe Carell Jr. Children's Hospital at Vanderbilt, Nashville, Tennessee, USA; 11 Stanford University School of Medicine, Stanford, California, USA; 12 Children's Hospital of Pittsburgh, Pittsburgh, Pennsylvania, USA; 13 UC Irvine Medical Center, Orange, California, USA; 14 University of Turin, Turin, Italy; 15 Birmingham Children's Hospital NHS Foundation Trust, Birmingham, UK; 16 Cairo University Children's Hospital, Cairo, Egypt; 17 Hospital for Sick Children, Toronto, Ontario, Canada; 18 University of Minnesota, Minneapolis, Minnesota, USA; 19 Ain Shams University Hospital, Cairo, Egypt

**Keywords:** cholesteryl ester storage disease, infants, lysosomal acid lipase deficiency, natural history, Wolman disease

## Abstract

**Purpose:**

The purpose of this study was to enhance understanding of lysosomal acid lipase deficiency (LALD) in infancy.

*Genet Med* **18** 5, 452–458.

**Methods:**

Investigators reviewed medical records of infants with LALD and summarized data for the overall population and for patients with and without early growth failure (GF). Kaplan–Meier survival analyses were conducted for the overall population and for treated and untreated patients.

*Genet Med* **18** 5, 452–458.

**Results:**

Records for 35 patients, 26 with early GF, were analyzed. Prominent symptom manifestations included vomiting, diarrhea, and steatorrhea. Median age at death was 3.7 months; estimated probability of survival past age 12 months was 0.114 (95% confidence interval (CI): 0.009-0.220). Among patients with early GF, median age at death was 3.5 months; estimated probability of survival past age 12 months was 0.038 (95% CI: 0.000-0.112). Treated patients (hematopoietic stem cell transplant (HSCT), *n* = 9; HSCT and liver transplant, *n* = 1) in the overall population and the early GF subset survived longer than untreated patients, but survival was still poor (median age at death, 8.6 months).

*Genet Med* **18** 5, 452–458.

**Conclusions:**

These data confirm and expand earlier insights on the progression and course of LALD presenting in infancy. Despite variations in the nature, onset, and severity of clinical manifestations, and treatment attempts, clinical outcome was poor.

*Genet Med* **18** 5, 452–458.

## Introduction

Lysosomal acid lipase deficiency (LALD; OMIM 278000; ref. 1) is an autosomal-recessive disorder caused by mutations of the *LIPA* gene located on chromosome 10q23.2-q23.3.^2^ Lysosomal acid lipase (LAL) is essential for the breakdown of cholesteryl esters and triglycerides in the lysosomes, and deficiency of LAL results in accumulation of these lipids in the tissues of affected individuals.^1,3^ The disease presents across an age continuum, from infants to adults, with common features including hepatomegaly, elevated serum transaminase concentrations, and progressive liver fibrosis and cirrhosis.^4,5,6,7^ In infants, growth failure (GF) and rapidly progressive hepatic disease are prominent features of LALD (historically known as Wolman disease) and key contributors to mortality, with death typically occurring within the first 6 months of life.^2,8,9,10^

Clinical familiarity with LALD is low, and many aspects of its clinical presentation, progression, and medical management are poorly understood. In addition, systematic analysis of the presentation of LALD in infants is lacking; current descriptions are largely based on case reports and small case series, many from more than 30 years ago.^5,6,7,11,12^ Medical records of infants with confirmed LALD were systematically reviewed to develop a more detailed understanding of the clinical manifestations and course of LALD presenting in infancy, including characterization of patient survival, to provide data to serve as a historical reference for efficacy studies of this population.

## Materials and Methods

### Study design, patients, and data collection

Initial study site selection was based on the completion of a feasibility questionnaire sent to ~500 physicians in 44 countries. A total of 154 physicians responded, and all physicians identified as being involved in the care of patients with LALD were considered for participation; site selection was based on willingness to participate.

The study protocol and other documents were reviewed by an institutional review board or equivalent body before study site initiation, and informed consent was obtained if required by local regulations. Eligibility for inclusion required a clinical diagnosis of LALD within the first 2 years of life―confirmed by either LAL enzyme activity testing or *LIPA* gene mutational analysis―and the availability of the following in their medical records: date of birth; sex; date of death or age at time of death if deceased; weight at birth (or first recorded weight) plus at least one weight obtained ≥4 weeks later but before initiation of any treatment with curative intent; test date, result, and name of testing center for LAL enzyme activity or *LIPA* gene mutational analysis; and date of initiation of hematopoietic stem cell transplant (HSCT) or liver transplant procedure, if applicable (defined as the first day of any pretransplant conditioning, if available). Additional data collected when available included method of diagnosis, demographics, clinical and family history, anthropometric measurements, physical examination findings, clinical chemistry results (including assessments of adrenal function, hematologic tests, and CD4-to-CD8 ratio), liver biopsy data, imaging findings, details of any supportive interventions (e.g., nutritional support including use of parenteral nutrition, blood transfusions), and autopsy results.

### Data analysis

Enrollment of approximately 40 patients with LALD presenting in infancy (i.e., before age 2 years) was expected to provide a reasonable description of the clinical presentation and outcomes of this rare disease.^13^ Continuous end points were summarized as the number of patients with nonmissing values, mean, SD, minimum, maximum, median, first quartile, and third quartile. Categorical end points were summarized as frequencies and/or using shift tables. Anthropometric data, including weight, length, head circumference, weight for length, and body mass index, were standardized to *z*-scores according to age/sex-normative data gathered by the World Health Organization^14,15^ and summarized as continuous data. Percentiles were computed from the *z*-scores. Anthropometric data were also represented as the number and percentage of patients meeting criteria for being underweight, defined as ≤2 SDs below the median weight for age. With the exception of survival data, data for patients who received a transplant were summarized only through the date of initiation of any pretransplant conditioning regimen, if available; otherwise, the date of transplant was used.

Because of the prominence of GF in the first 6 months of life in patients with LALD and the plausible link between this aspect of the disease and mortality, analyses were performed for the overall population and for the subset of patients who fulfilled defined early GF criteria similar to those used in an ongoing clinical trial of enzyme replacement therapy in infants with LALD (www.clinicaltrials.gov identifier NCT01371825). Early GF was defined as fulfillment of one or more of the following criteria before age 6 months: (i) decreased body weight across ≥2 of the 11 major percentiles on a standard World Health Organization weight-for-age chart; (ii) body weight (in kilograms) below the 10th percentile on a standard World Health Organization weight-for-age chart and no weight gain for the previous 2 weeks; and (iii) loss of ≥5% of birth weight in children >2 weeks of age. As appropriate, summary statistics and statistical analyses also were performed for treated (i.e., those who received liver transplant or HSCT) and untreated patients.

Time from birth to death and proportions of patients alive at selected ages were estimated using Kaplan–Meier methodology. Estimates of the median (with exact 95% confidence intervals) and upper and lower quartiles of time to death were derived and plotted. Time-to-event analyses were conducted for all eligible patients with evaluable data and for the subsets of patients with and without early GF. For each analysis, a standard life table of time to death was created for the overall population and for treated (i.e., received transplant) and untreated patients. Time to death in treated and untreated patients was compared using log-rank tests. A multivariate Cox proportional hazard regression analysis model was used to examine the effect of the following covariates on survival: sex, country of origin, transplant, blood transfusion, enteral supplementation, parenteral supplementation, and steroid therapy. No data imputation was planned or conducted.

## Results

### Population

Forty patients who received a clinical diagnosis of “Wolman disease” between 1985 and 2012 were enrolled. Four were ineligible because they lacked a confirmed diagnosis (*n* = 2) or a second weight measurement (*n* = 2). In addition, one patient was excluded from this analysis because he was later enrolled in a separate clinical study.

There were 35 patients in the overall population, enrolled from 17 centers in 6 countries. Of these patients, 26 were classified as having early GF. Baseline demographic characteristics and clinical/family histories are shown in **Table 1**. Most patients (70%) who received transplant were from the United States. LALD was diagnosed by enzyme activity testing in 34 patients (97%). Homozygous mutations were reported for 3 of the 12 patients who had *LIPA* mutation analysis performed. Two siblings (twins) had identical null mutations arising from a premature stop codon (TAT>TAG) at exon 298; all other patients had unique mutations. No patients had a copy of the exon 8 splice junction mutation (c.894G>A).

### Symptom onset and disease presentation

Overall, 43% of patients had first symptom onset before the age of 1 month. Median ages at first symptom onset were similar for the subsets with and without early GF (1.1 month (range: 0.0–3.0 months) and 1.0 month (range: 0.23–6.0 months), respectively). Prominent manifestations included vomiting, diarrhea, and steatorrhea, accompanied by liver biochemical abnormalities. The median time interval between symptom onset and diagnosis was 1.2 months (range: 0.05–17.0 months). The median age at diagnosis was lower among patients with early GF than among those without early GF (2.5 months (range: 1.0–5.0 months) and 5.0 months (range: 1.0–17.7 months), respectively).

### Growth failure

Weight-for-age *z*-scores were low before diagnosis (overall: mean, −2.09; median, −2.64; early GF: mean, −1.98; median, −2.55) and worsened with disease progression, decreasing from the first record to death by a mean of −1.98 (median: −1.75) in the overall population and by a mean of −2.40 (median: −2.72) in the early GF subgroup. The percentage of underweight patients increased over time in the overall population (20, 54, and 66% at first record, diagnosis, and death, respectively).

### Liver biochemical findings

**Figure 1:**
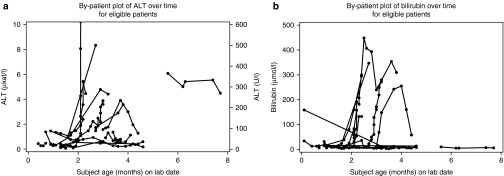
Spaghetti plot of (**a**) alanine aminotransferase (ALT) and (**b**) bilirubin changes over time in infants with lysosomal acid lipase deficiency.

Median alanine aminotransferase (ALT) and aspartate aminotransferase (AST) concentrations in the overall population were elevated at diagnosis (56.5 U/l (0.94 μkat/l; *n* = 24) and 151 U/l (2.52 μkat/l; *n* = 19), respectively). At diagnosis, ALT concentration was abnormal in 14 of 24 patients, including 8 patients who had ALT values more than three times the upper limit of normal. Median ALT values substantially increased with disease progression in patients with and without early GF (**Figure 1a**). Median ALT concentration at death (or last measurement) in the overall population was 110.5 U/l (1.85 μkat/l; range: 13–851 U/l (0.22–14.21 μkat/l); *n* = 20). Similarly, AST concentrations were elevated at diagnosis in 18 of 19 patients, and AST concentrations increased with disease progression. Median AST concentration at death (or last measurement) was 283 U/l (4.73 μkat/l; range: 35–4,250 U/l (0.58–70.97 μkat/l); *n* = 26). γ-Glutamyl transferase concentrations were elevated at diagnosis in 11 of 13 patients, and γ-glutamyl transferase concentrations increased after diagnosis in 10 of 12 patients with available values. Total bilirubin concentrations at diagnosis were elevated in 11 patients (52%), of which 8 patients had bilirubin more than three times the upper limit of normal; the other 10 patients had bilirubin concentrations that were normal (*n* = 9) or below normal (*n* = 1). Worsening of bilirubin concentrations over time was noted in some patients, and no patient shifted to a less severe bilirubin category (**Figure 1b**).

Of the four patients who had unique prothrombin time measurements at both diagnosis and death, the median change was 1.2 s (range: −3.6 to 2.8 s). Of the four patients who had unique partial thromboplastin time measurements at both diagnosis and death, the median change was 7.0 s (range: 0.4–39.9 s).

Median serum albumin concentration was low at diagnosis (26.0 g/l; range: 15–36 g/l; *n* = 22). In the eight patients who had unique serum albumin measurements both at diagnosis and at death, the median change was −0.5 g/l (range: −9 to 21 g/l).

### Pathology findings

Liver biopsies were performed in nine patients at a median age of 5.2 months (range: 1.5–43.9 months). All nine patients had evidence of fibrosis (*n* = 6) and/or steatosis/lipid vacuoles (*n* = 6). Autopsy reports were available for five patients. Liver enlargement and spleen enlargement were evident in four and five patients, respectively; reports included yellow-orange discoloration, fatty infiltration/steatosis, necrosis, fibrosis, and cirrhosis. Adrenal abnormalities, including enlargement, necrosis, and microcalcifications, were noted in all five cases.

### Lipid biochemistry

Median total cholesterol concentrations were normal at diagnosis (2.99 mmol/l; range: 1.54–4.58 mmol/l; *n* = 16). Marked hypercholesterolemia (total cholesterol >5.17 mmol/l) occurred in two patients who did not have early GF, showed no clinical evidence of failure to thrive, and survived >12 months before receiving transplant. Median triglyceride concentration was elevated at diagnosis (2.43 mmol/l; range: 1.3–6.0 mmol/l; *n* = 15). High-density lipoprotein cholesterol levels were low at diagnosis, with 67% of patients having high-density lipoprotein cholesterol values <50% of the lower limit of normal at diagnosis.

### Imaging findings

Among 34 patients who had at least one abdominal imaging procedure, 27 (79%) had adrenal calcification (including 15 on X-ray). Hepatomegaly and/or splenomegaly were noted in 27 patients.

### Supportive interventions and other treatments

Thirty-three patients (94%) in the overall population received a supportive intervention, which most commonly included nutritional support (*n* = 25, 71%) or blood transfusions (*n* = 22, 63%). Blood transfusions were reported by a similar proportion of patients with early GF and those without early GF. Nutritional support most often involved the administration of enteral (46%) or parenteral (49%) supplements. Steroid therapy was administered to five patients (14%), though there was no evidence of adrenal insufficiency per an adrenal function test.

Ten patients (29%) received HSCT. The median age at treatment was 5.4 months (range: 2.2–45.0 months), with most (7/10) patients receiving treatment before age 12 months. Five patients with early GF received treatment, all within the first 8 months of life. Only three patients had HSCT after age 12 months (at ages 19, 35, and 45 months). One patient who received a bone marrow transplant at age 35.1 months and died at age 37.3 months had received a liver transplant at age 4.1 months.

### Survival

**Figure 2:**
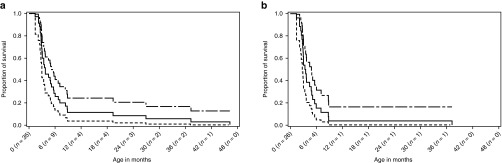
Kaplan–Meier plot of time from birth to death in the (**a**) overall population and (**b**) infants with early growth failure. Top line is “Upper 95% Confidence Limit”, Middle line is “Survival Function Estimate” and bottom line is “Lower 95% Confidence Limit”.

Although the protocol allowed for enrollment of living patients, all patients in the overall population were deceased at enrollment. The median age at death was 3.7 months (range: 1.4–46.3 months), and the Kaplan–Meier estimate for the probability of survival past age 6 months was 0.257 (**Figure 2a**, **Table 2**). Only four patients (one with early GF, three without early GF) survived beyond age 12 months. Two of these patients had undergone HSCT (surviving to ages 46.3 and 26.9 months) and one underwent liver transplant and HSCT (surviving to age 37.3 months). The remaining patient (without early GF) was untreated and survived to age 19.3 months. The Kaplan–Meier estimate for probability of survival past age 12 months was 0.114 (**Figure 2a**, **Table 2**). Log-rank test results suggested that, although all patients died prematurely, treated patients survived significantly longer (*P* < 0.001; median age at death, 8.6 months; range: 2.6–46.3 months) than did untreated patients (median age at death: 3.0 months; range: 1.4–19.3 months).

Among patients with early GF, the median age at death (3.5 months; range: 1.4–37.3 months) was similar to that of the overall population; however, Kaplan–Meier estimates for survival to ages 6 and 12 months were lower (**Figure 1b**, **Table 2**). This difference was largely attributable to the long survival duration of three patients without early GF, including one untreated patient who survived to 19.3 months and two treated patients who survived to ages 26.9 months and 46.3 months. Patients with early GF who received transplant had modest but significantly longer survival than did untreated patients with early GF (*P* = 0.008). Among untreated patients with early GF, the probability of survival past age 6 months was lower still, and an estimated zero probability of survival past age 12 months was predicted in this group (**Table 2**). Kaplan–Meier survival estimates in patients without early GF were greater than those of the overall population, and survival did not differ significantly between treated and untreated patients (*P* = 0.14).

In a Cox proportional hazard regression analysis model for the overall population, country of origin, treatment, enteral supplementation, and parenteral supplementation were associated with reduced risk of death (**Supplementary Table S1** online).

## Discussion

These data confirm and expand earlier insights on the rapidly progressive and fatal course of LALD presenting in infants and provide a more comprehensive understanding of disease progression and factors that might influence the disease course. Clinical presentation with diarrhea, vomiting, abdominal distension, and hepatosplenomegaly was common early in life and is consistent with previous descriptions of LALD in infants.^4,5,6,7^ Undernourishment with loss of subcutaneous fat and muscle was noted as a prominent feature in early case reports of infants with LALD,^5,7^ and a review of published cases performed before initiation of this study suggested that early GF might be a sensitive and specific marker for risk of rapid disease progression and early death. In this study weight-for-age *z*-scores were low at birth/first record and worsened with disease progression. Although only 20% of patients were underweight at birth/first record, >50% of patients were underweight by the time of diagnosis (median age at diagnosis: 2.6 months). These findings confirm that early GF is a prominent manifestation of LALD presenting during infancy.

Liver biochemical abnormalities were present early in the disease course, with 58 and 95% of patients having elevations in ALT and AST concentrations at diagnosis, respectively. Some patients showed marked, rapid, progressive increases in serum transaminase concentrations over time, accompanied by other indicators of progressive liver injury and dysfunction, including elevated total bilirubin concentrations, abnormal coagulation values, and depressed serum albumin concentrations. These findings suggest that rapidly progressive liver disease with hepatocellular failure is likely an important contributor to overall mortality in infants presenting with LALD.

Consistent with previously published findings,^5,6,7^ fibrosis and lipid accumulation in the liver were common in this study. Fibrosis was noted in six of the nine patients who underwent liver biopsy and was found in four of these patients before age 6 months and in one patient as early as age 1.5 months. The limited number of biopsies may reflect the risk associated with the liver biopsy procedure in these critically ill infants. In addition, although rapidly progressive liver disease contributes to mortality in infants with LALD, the complications of portal hypertension and esophageal varices that often occur in children and adults with LALD^4^ were not prominent in this study, which may relate to the rapidity of disease progression in infants.

In this study total cholesterol levels were generally normal, and, although triglyceride concentrations were elevated in most patients with available data, these abnormalities were generally twice the upper limit of normal or less. Although there was evidence of low high-density lipoprotein cholesterol levels in this study, data on low-density lipoprotein cholesterol levels were very limited but did not show marked abnormalities. These findings are consistent with the lipid abnormalities described in previous reports.^5,11,16^

The prognosis for infants presenting with LALD is extremely poor. Median age at death was 3.7 months, inclusive of patients receiving HSCT, and all but four patients were deceased by age 9 months. For the overall population, the Kaplan–Meier estimate of the probability of survival past age 12 months was 0.114, and transplant was associated with only modest improvement in survival; median ages at death were 8.6 months in treated patients and 3.0 months in untreated patients. Clinical outcome was particularly poor among untreated patients with early GF, for whom the Kaplan–Meier estimate for probability of survival past age 6 months was 0.048 and zero probability of survival beyond age 12 months was predicted. Notably, the median age at treatment (5.4 months) exceeded the median age at death in the overall population (3.7 months), which suggests a possible bias toward transplant in patients with less rapidly progressive disease.

Although survival beyond 3 years has been reported for three patients diagnosed with Wolman disease who received HSCT,^17,18^ most HSCT attempts have resulted in early death.^8,18,19,20^ In infants, LALD is a progressive disease and usually fatal before 6 months of age, with or without active treatment. Liver complications in infants with LALD resemble those seen in affected children and adults, with hepatomegaly, elevated transaminase concentrations, liver steatosis, and fibrosis. This comprehensive characterization of the presentation and clinical course of LALD in infants should prove valuable to clinicians, could help prevent diagnostic delays, and may serve as a historical control for studies of emerging therapeutic interventions.

## Disclosure

M. Banikazemi's institution has received grant money from Synageva for clinical trial support. M.Bialer's institution has received grant money from Synageva for clinical trial support. A.C.'s institution has received a study grant from Synageva for clinical trial support. A.D. has received consulting/honorarium fees from Synageva and Cytonet. M.D.R. has received payment for board membership from Genzyme, a Sanofi company, and lecture fees from Genzyme, a Sanofi company; Shire; BioMarin; and Actelion. S.E. and E.S. are former employees of Synageva and have received stock/stock options from Synageva. D.F. is an employee of the University of Pittsburgh and has received stock/stock options from DiaVacs, and his institution has received grant money from various federal and nonfederal sources. J.J.G.'s institution has received grant money from Synageva. O.G. has previously received travel support and payment for board membership and for development of educational presentations from Synageva. C.H.'s institution has received travel support from Synageva. S.J. has received consulting fees and travel support from Synageva, and he and his institution have received consulting fees from Genzyme, Shire, BioMarin, and Ultragenyx; his institution has received grant money from Shire. A.G.Q. is an employee of Synageva and has received stock/stock options from Synageva. J.R. has received payment for advisory board membership and travel support from Genzyme, Shire, BioMarin, Actelion, and Allexion, as well as lecture fees from Genzyme, Shire, BioMarin, and Actelion. His institution has received unrestricted educational grants from Genzyme, Shire, BioMarin, and Actelion. E.S. is a former employee of Synageva and has received stock/stock options from Synageva. V.V. has received lecture fees, payment for board membership, and funding for clinical trials from Synageva, and his institution has received grant money from Synageva. C.W.'s institution has received grant money from the University of Minnesota and travel support from Synageva. S.C., J.D., G.M.E., I.G.M., L.A.S., and O.Z. have nothing to disclose.

## Figures and Tables

**Table 1:**
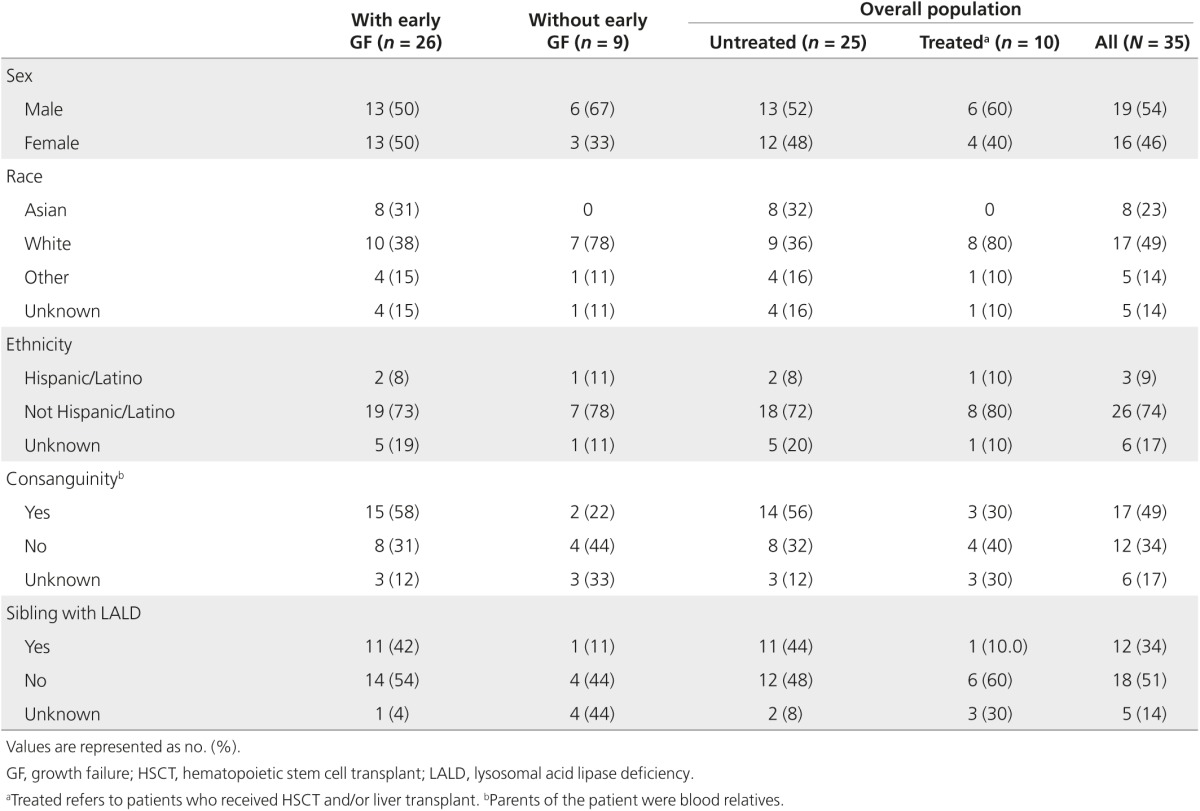
Baseline demographic characteristics and clinical/family history

**Table 2:**
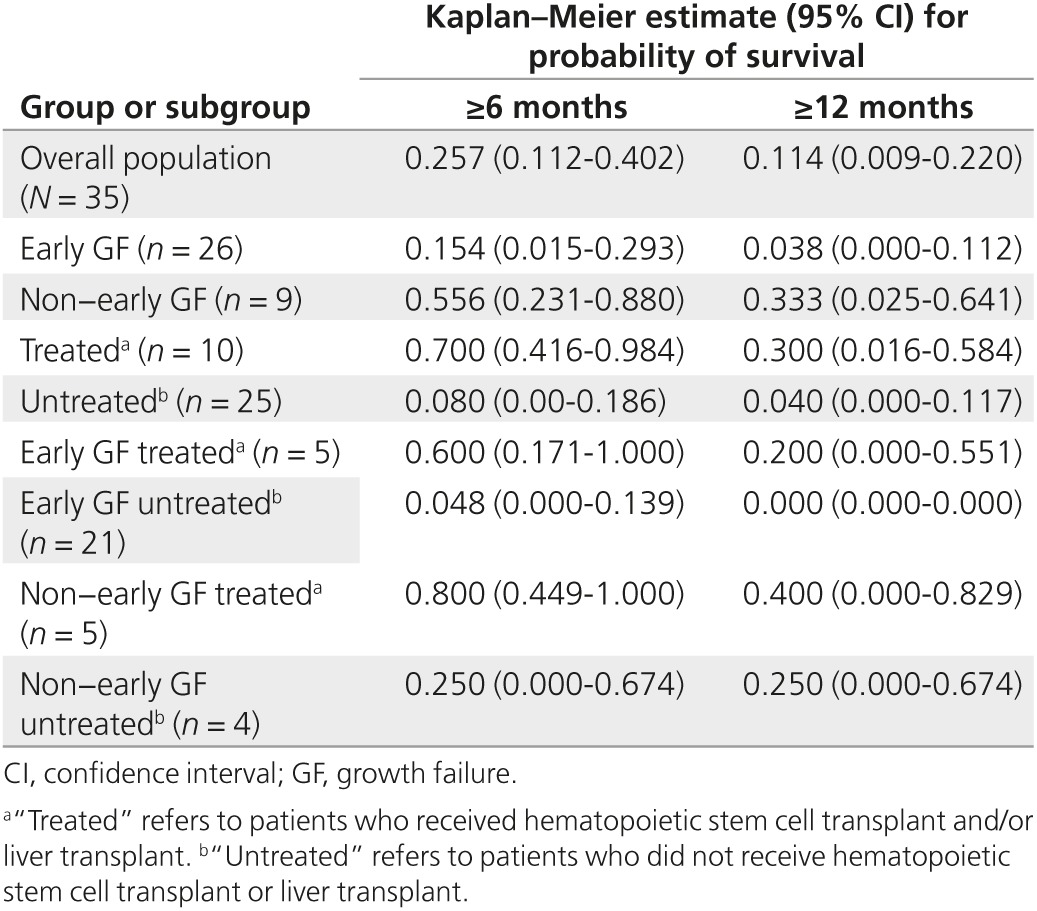
Survival estimates by population subgroups
