# Supplementary material for: Rapid progression and mortality of lysosomal acid lipase deficiency presenting in infants
Source: Genet Med. 2015 Aug 27;18(5):452–8. doi: 10.1038/gim.2015.108 (PMC4857209; doi:10.1038/gim.2015.108)
Supplement: Supplementary Table S1 [file gim2015108x1.doc]

**Supplementary Table 1.** Summary of Hazard Ratio for Multivariate Cox Proportional Hazard Analyses of Survival

**(Tables 41.1, 41.1.1, 41.2, 41.2.1)**

|  | **Patients With Early GF** | | **Overall Population** | |
| --- | --- | --- | --- | --- |
|  | **Untreated (n=21)** | **All (n=26)** | **Untreated (n=25)** | **All (n=35)** |
| Female vs male | 1.67 (0.43, 6.51) [0.46] | 1.42 (0.46, 4.38) [0.54] | 0.86 (0.25, 2.98) [0.81] | 1.56 (0.55, 4.40) [0.40] |
| Country of origin*^a^* | | | | |
| US vs Canada | 2.60 (0.31, 21.97) [0.38] | 4.02 (0.46, 35.15) [0.21] | 7.20 (1.13, 45.92) [0.04] | 4.31 (0.96, 19.41) [0.06] |
| US vs Egypt | 20.39 (0.77, 540.80) [0.07] | 33.49 (1.27, 884.80) [0.04] | 61.53 (2.69, 1407.47) [0.010] | 38.35 (2.39, 614.69) [0.010] |
| US vs France | 0.12 (0.01, 1.94) [0.13] | 0.50 (0.04, 6.58) [0.60] | 0.60 (0.07, 5.10) [0.64] | 0.90 (0.16, 4.87) [0.90] |
| US vs. Italy | NA | NA | 0.86 (0.09, 8.26) [0.90] | 0.61 (0.09, 4.40) [0.63] |
| US vs UK | 2.04 (0.27, 15.53) [0.49] | 3.12 (0.41, 23.45) [0.27] | 4.57 (0.73, 28.67) [0.10] | 2.87 (0.77, 10.68) [0.12] |
| Treatment*^b^* | NA | 5.72 (0.62, 52.95) [0.12] | NA | 6.12 (1.62, 23.08) [0.008] |
| Blood transfusion | 4.49 (0.97, 20.76) [0.05] | 2.27 (0.67, 7.74) [0.19] | 2.20 (0.55, 8.83) [0.26] | 2.61 (0.84, 8.07) [0.10] |
| Enteral supplement | 0.28 (0.07, 1.10) [0.7] | 0.23 (0.06, 0.83) [0.02] | 0.18 (0.05, 0.71) [0.01] | 0.23 (0.07, 0.74) [0.01] |
| Parenteral supplement | 0.32 (0.08, 1.19) [0.09] | 0.30 (0.09, 1.05) [0.06] | 0.24 (0.07, 0.89) [0.03] | 0.26 (0.09, 0.78) [0.02] |
| Steroid therapy | 1.17 (0.15, 9.03) [0.88] | 1.99 (0.40, 10.01) [0.40] | 2.07 (0.34, 12.53) [0.43] | 2.96 (0.78, 11.47) [0.11] |
| Abbreviations: GF, early growth failure; HSCT, hematopoietic stem cell transplant; NA, not applicable.  Data are shown as hazard ratio (95% CI) [*P* value]. *^a^* Analyses by country of origin used the location of the study site where the patient was enrolled, as this was typically (but not always) the location where the patient was treated). *^b^* Treatment refers to HSCT and/or liver transplant. | | | | |
